# Intraoperative cricothyroid muscle electromyography may contribute to the monitorization of the external branch of the superior laryngeal nerve during thyroidectomy

**DOI:** 10.3389/fendo.2023.1303159

**Published:** 2023-12-07

**Authors:** Nurcihan Aygun, Mehmet Taner Unlu, Mehmet Kostek, Ozan Caliskan, Adnan Isgor, Mehmet Uludag

**Affiliations:** ^1^ General Surgery Department, Sisli Hamidiye Etfal Training and Research Hospital, Istanbul, Türkiye; ^2^ General Surgery Department, Memorial Sisli Hospital, Sisli, Istanbul, Türkiye

**Keywords:** cricothyroid muscle, electromyogarphy, intraoperative neural monitoring (IONM), external branch of the superior laryngeal nerve (EBSLN), thyroidectomy

## Abstract

**Background:**

In thyroid surgery, both the recurrent laryngeal nerve (RLN) and external branch of the superior laryngeal nerve (EBSLN) should be preserved for maintaining the vocal cord functions. We aimed to evaluate whether EMG of the CTM applied after the superior pole dissection provided additional informative data to the IONM via ETT or not, regarding the EBSLN function.

**Methods:**

The prospectively collected data of the patients, who have undergone thyroidectomy with the use of IONM for the exploration of both the RLN and EBSLN between October 2016 and March 2017, were evaluated retrospectively. Patients over 18 years of age with primary thyroid surgery for malignant or benign thyroid disease, and whom were applied CTM EMG with a needle electrode after the completion of thyroidectomy were included in the study. In the study, each neck side was evaluated as a separate entity considering the EBSLN at risk.

**Results:**

The data of 41 patients (32 female, 9 male) (mean age, 46.7 + 9.1; range, 22-71) were evaluated. Sixty seven EBSLNs out of 26 bilateral and 15 unilateral interventions were evaluated. With EBSLN stimulation after the superior pole dissection, positive glottic EMG waveforms via ETT were obtained in 45 (67.2%) out of 67, and the mean glottic amplitude value was 261 + 191 μV (min‐max: 116‐1086 μV). Positive EMG responses via the CTM EMG were achieved from all of the 67 EBSLNs (100%) with stimulation using a monopolar probe at the most cranial portion above the area of divided superior pole vessels. The mean value of CTM amplitudes via CTM EMG obtained with EBSLN stimulation was 5268 + 3916 μV (min‐max:1215 ‐19726 μV). With EBSLN stimulation, the mean CTM EMG amplitude was detected significantly higher than the mean vocal cord amplitude (p<0.0001). The CTM EMG provided more objective quantifiable data regarding the EBSLN function (100% vs 67,2%, p<0.001).

**Conclusion:**

In addition to the IONM via ETT, intraoperative post-dissection CTM EMG via needle electrode is a safe, simple and applicable method that may provide significant additional informative data to IONM with ETT by obtaining and recording objective quantitative data related to the EBSLN function.

## Introduction

In thyroid surgery, both the recurrent laryngeal nerve (RLN) and external branch of the superior laryngeal nerve (EBSLN) should be preserved to maintain vocal cord functions (1). The adductor muscles (thyroarytenoid muscle (TAM) (main adductor muscle), lateral cricoarytenoid muscle, and interarytenoideus muscle) and abductor muscle (posterior cricoarytenoid muscle) of the larynx are supplied by the motor fibers of the RLN, while the tensor muscle of the vocal cords, cricoarytenoid muscle (CTM), is innervated by the EBSLN (2).

After innervating the CTM, the EBSLN enters the larynx extending through the cricothyroid membrane and makes anastomosis with the RLN in one-third anterior part of the TAM in the larynx, hence contributing to its motor innervation. This anastomosis is called the “human communicating nerve” (3). This nerve has been reported in up to 85% of anatomical dissection studies (3, 4).

The EBSLN has a highly variable course toward the CTM and is in a close anatomical relationship with the superior thyroid vessels. Therefore, it is a potential risk during superior pole dissection during thyroid surgery. Different classifications have been defined regarding EBSLN anatomy, and the Cernea classification is the most widely used classification system. The Cernea classification, based on the risk of EBSLN injury in thyroidectomy, categorizes the nerve according to its relation to the superior thyroid vessels and the upper edge of the superior thyroid pole. In the Cernea classification, the EBSLN crosses with the superior thyroid vessels at a distance more than 1 cm cranially from the upper edge of the superior pole in type I, less than 1 cm from the upper edge of the superior pole in type IIa, and below the upper edge of the superior pole in type IIb (5). Although both type IIa and type IIb nerves have a risk of injury during dissection and ligation of the superior thyroid vessels due to their close course to the superior pole, type IIb has the highest risk. In the last meta-analysis, the prevalences of type I, type IIa, and type IIb in intraoperative studies were reported as 35.23 (95% CI 26.07–42.57), 44.27% (95% CI 34.34–51.55), and 20.50% (95% CI 13.32–27.25), respectively (6).

Recently, the usage of intraoperative neuromonitorization (IONM) during thyroid surgery has increased considerably (7). IONM via endotracheal tube (ETT) with surface electrodes is a widely accepted technique as an aid to the gold standard of visual identification, which can be used for both the identification and assessment of the functions of the RLN and EBSLN (8, 9). In order to minimize the potential risk of EBSLN injury, three basic techniques have been described during superior pole dissection, including division of the superior pole vessels on the thyroid capsule without exploring the EBSLN, visual identification of the EBSLN before the division of the vessels, or mapping of the nerve using IONM or a stimulator probe during superior pole dissection (9–11). Although there is no consensus about which of these methods is the best method for the protection of the EBSLN, it has been reported that the use of IONM improves the visual and functional identification of the EBSLN and may reduce the risk of EBSLN injury (12–14).

The true positivity of EBSLN stimulation with IONM is defined as contraction in the CTM, which is the target organ of the EBSLN, and/or recording of endotracheal glottic electromyography (EMG) waveform obtained via ETT (9). The main target organ of the EBSLN is the CTM, and the CTM twitch with EBSLN stimulation is not a recordable quantitative data. Although it was reported in a study that positive EMG response (positive threshold value >50 μV) with EBSLN stimulation was recordable in 100% via the reinforced endotracheal tube with surface electrodes, in other studies, 68%–80% positive response (positive threshold value >100 μV) was obtained with EBSLN stimulation using standard ETT with surface electrodes (13–16).

Although the glottic EMG response is quantitative data, this finding is not related to the CTM motor function, which is mainly innervated by the EBSLN. This EMG activity is related to the adductor function of the vocal cord and motor innervation provided by the connection between the RLN and the EBSLN in the anterior third of the TAM described above as a “human communicating nerve”. Although the rate of positive glottic EMG response has been found similar to that of human communicating nerve reported in human anatomical studies, the relationship of the magnitude of the EMG amplitude with the function of the EBSLN is yet to be clarified (9). In addition, there is no quantitative glottic EMG response in at least 20% of nerves.

Traditionally, RLN and EBSLN injuries, well-known complications of thyroidectomy, have been considered the main cause of postoperative voice problems. However, voice disorders are common after thyroidectomy, many of which are not associated with laryngeal nerve injuries and are multifactorial. More recently, many factors have been suggested such as arytenoid cartilage problems due to intubation, changes in laryngeal vascularization and lymphatic drainage due to surgery, cricothyroid and strap muscle injuries, injury to the anastomotic branches in the perithyroidal neural plexus, laryngotracheal fixation causing deficiency in vertical movement of the larynx, local pain in the neck, and development of psychological reaction (1).

Voice disorders after thyroidectomy might be a great concern for patients as well as complications such as hypocalcemia and cervical scarring (17). Postthyroidectomy vocal disorders can negatively affect the work performance of professionals such as singers, announcers, lawyers, and teachers, who use their voices. However, with the increasing awareness of the quality of life recently, the importance of postthyroidectomy voice disorders in terms of quality of life is increasing for all patients (18).

Quantitative recording of intraoperative nerve functions may be important in the differential diagnosis of postoperative voice disorders as well as the preservation of laryngeal nerves in thyroidectomy. In addition, recording may provide more protection against unfounded claims related to sound problems in malpractice cases (19).

Intraoperative CTM EMG via a needle electrode may provide additional quantitative data regarding the EBSLN function. We aimed to evaluate whether the EMG of the CTM applied after superior pole dissection provided additional informative data to the IONM via ETT or not regarding the EBSLN function.

## Materials and methods

The prospectively collected data of the patients who have undergone thyroidectomy with the use of IONM for the exploration of both the RLN and EBSLN between October 2016 and March 2017 were evaluated retrospectively. Patients over 18 years of age with primary thyroid surgery for malignant or benign thyroid disease and who underwent CTM EMG with a needle electrode after the completion of thyroidectomy were included in the study.

In the study, each neck side was evaluated as a separate entity due to the EBSLN at risk. Patients with secondary thyroidectomy, with preoperative vocal cord paralysis (VCP), with lateral neck dissection, with divided cricothyroid muscle or EBSLN due to extrathyroidal extension, who were operated on without IONM due to technical problems, and who were under the age of 18 years were excluded.

### Surgical technique

The patients underwent thyroidectomy with the use of IONM. Thus, general anesthesia was applied using a low-dose short-acting neuromuscular blocking agent (rocuronium 0.3 mg/kg) only for the induction. They were intubated with a Nerve Integrity Monitor Standard Reinforced Electromyography Endotracheal Tube (size 6.0, 7.0, or 8.0) (Medtronic Xomed, Jacksonville, FL, USA). The surgical technique and the neuromonitoring method of the RLN and EBSLN have been described in detail in our previous studies (14, 20, 21).

### Intraoperative neuromonitorization

IONM of the RLN and EBSLN and intraoperative EMG of the CTM were performed using a four-channel NIM 3.0 Nerve Monitoring System (Medtronic Xomed, Jacksonville, FL, USA). A monopolar stimulator probe (Medtronic Xomed) was used for the nerve stimulation at a current of 1.0 mA with a duration time of 100 μs, a frequency of 4 Hz, and an event threshold of 100 μV. The setup, application, data collection, and interpretation of IONM were carried out in accordance with the International Neural Monitoring Study Group guidelines (8, 9). A standard four-step procedure (V1, R1, R2, and V2) was performed for the RLN. The EMG waveform amplitude achieved via RLN stimulation defined the motor function of the thyroarytenoid muscle, which is the main adductor muscle of the vocal cords. For the RLN and vagus, audible alarm of the system combined with an EMG amplitude of ≥100 μV was defined as positive stimulation (8).

The function of the EBSLN via IONM was evaluated with visual CTM twitch and positive glottic EMG response in case of its presence. Positive stimulation for the EBSLN was also defined as the EMG response of >100 μV and the auditory signal of the monitoring system. S1 was defined as the first response achieved with EBSLN stimulation at identification, and S2 was the final response achieved at the most proximal point of the EBSLN located above the ligated superior pole vessels (22).

### Superior pole dissection

**Figure 1:**
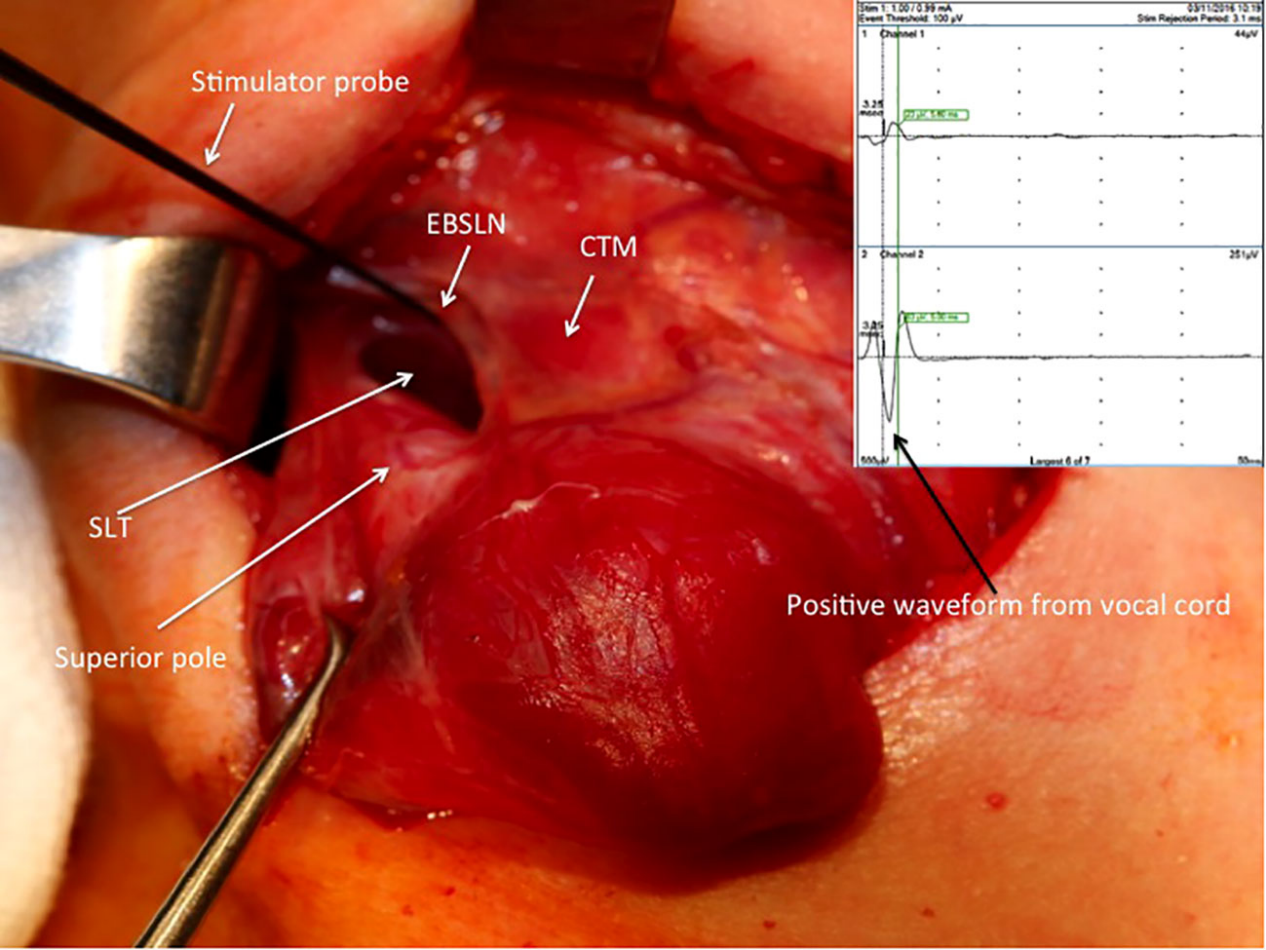
Positive EMG waveform achieved from the vocal cords with external branch of the superior laryngeal nerve stimulation using a stimulator probe during right superior pole dissection after the exposure of the sternothyroid-laryngeal triangle. SLT, sternothyroid-laryngeal triangle; EBSLN, external branch of the superior laryngeal nerve; CTM, cricothyroid muscle; EMG, electromyography.

**Figure 2:**
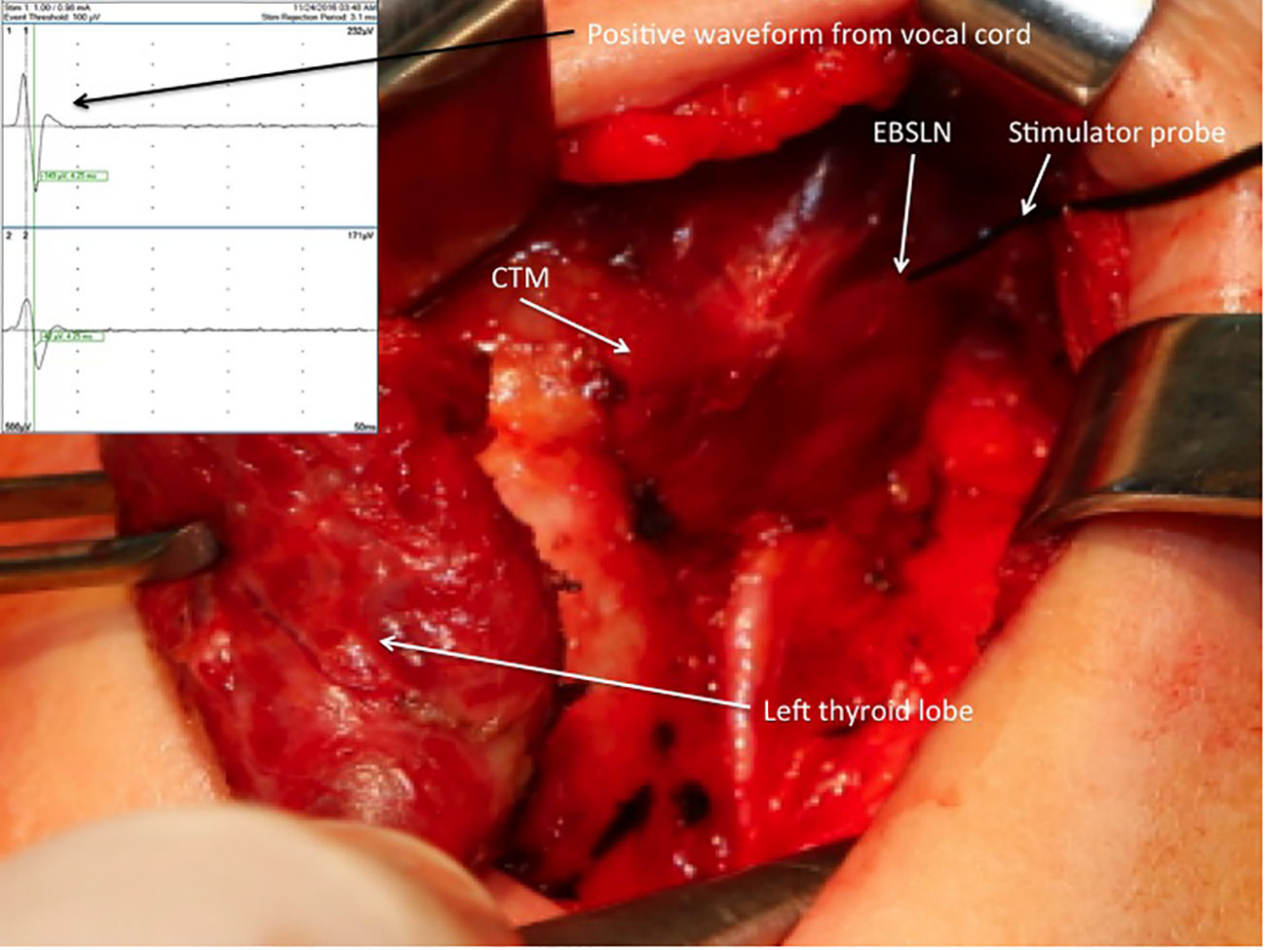
Positive EMG waveform achieved from the vocal cords with external branch of the superior laryngeal nerve stimulation using a stimulator probe at the most cranial portion above the ligated superior thyroid vessels after left superior pole dissection. EBSLN, external branch of the superior laryngeal nerve; CTM, cricothyroid muscle; EMG, electromyography.

After the vagus nerve was identified in the carotid sheath and initial vagal nerve stimulation (V1) was achieved, the superior pole was retracted inferolaterally for the dissection of the avascular plane and exposure of sternothyroid-laryngeal triangle (SLT), which harbors the EBSLN. The EBSLN was searched visually, and the fibrillar structure presumed to be the EBSLN was confirmed using a monopolar stimulator probe via CTM twitch evaluation. Additionally, if a positive glottic EMG response was achieved, it was recorded (S1) (**Figure 1**). If the EBSLN was not able to be identified visually and no CTM twitch was obtained with stimulation of any fibrillar structure using a probe, the CTM was directly stimulated using a probe, and the twitch was observed. Afterward, the EBSLN was searched using a probe in the area under the laryngeal insertion of the sternothyroid muscle and followed over the inferior pharyngeal constrictor (IPC) muscle toward the CTM. The fibers of the IPC muscle were not dissected thoroughly if the EBSLN was not able to be detected using the probe. Whether or not the EBSLN was identified, any of the dissected structures were not divided without stimulating it using a probe to confirm the absence of CTM twitch (21). The EBSLN was stimulated using a probe at the most cranial portion above the ligated superior thyroid vessels to confirm its integrity considering the CTM twitch and if there was also a positive glottic EMG response (S2) (**Figure 2**).

### Intraoperative cricothyroid muscle electromyography

**Figure 3:**
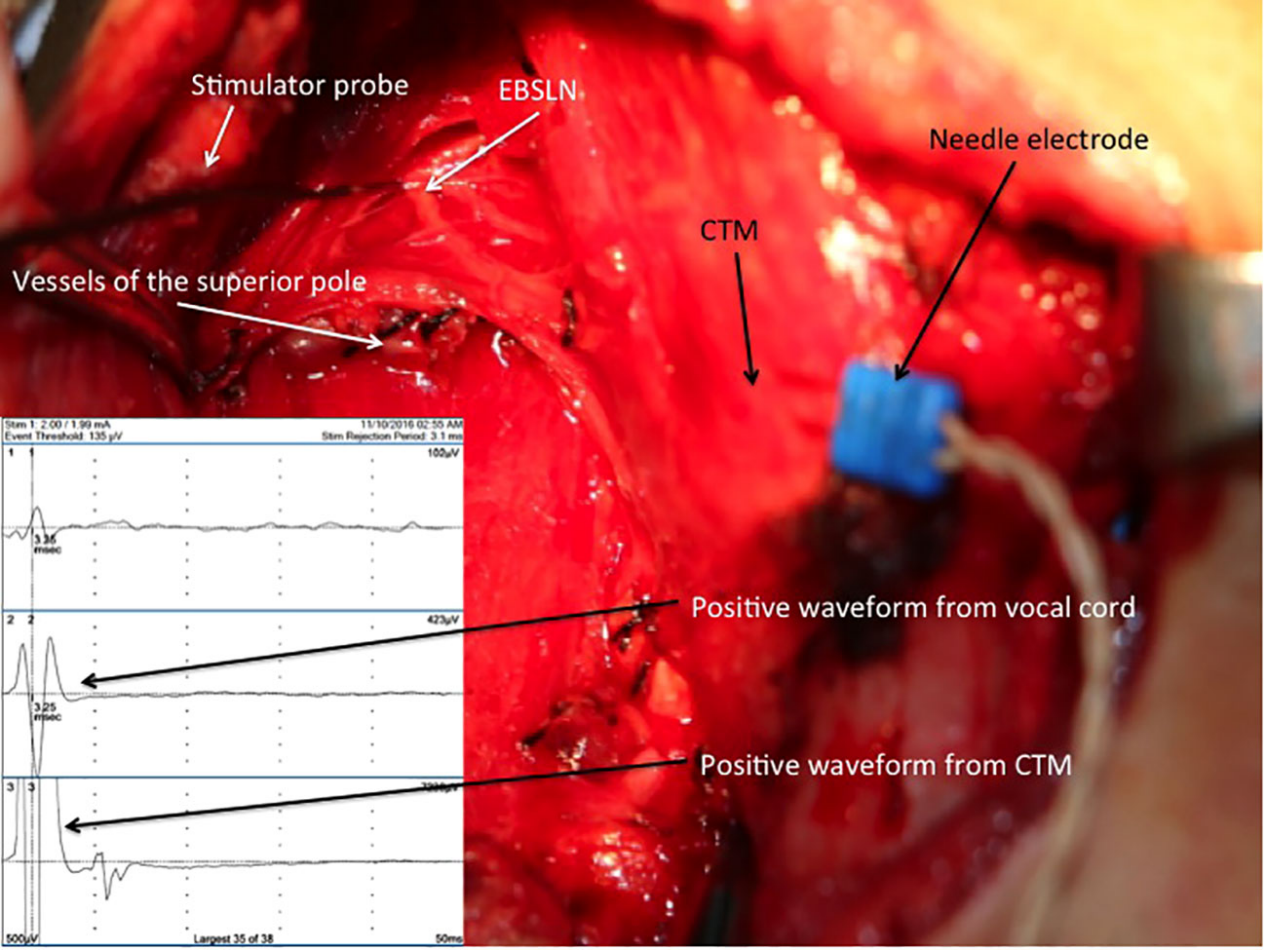
Positive EMG waveforms recorded from the vocal cords (channel 2) and CTM (channel 3) with external branch of the superior laryngeal nerve stimulation via a needle electrode inserted into the right CTM after the completion of thyroid resection. EBSLN, external branch of the superior laryngeal nerve; CTM, cricothyroid muscle; EMG, electromyography.

A pair of needle electrodes was inserted through the CTM, and it was plugged into the third channel of the interface connector box of the NIM 3.0 nerve monitoring system. The EBSLN was stimulated using a monopolar stimulator probe with a current of 1 mA at the most cranial portion above the divided superior thyroid vessels, and the EMG response was recorded (14) (**Figure 3**). As Martin-Oviedo et al. suggested in their study, positive CTM EMG response was accepted to be the amplitude achieved with EBSLN stimulation, which was at least four times greater than that of the uninnervated muscle (23). In our previous study, with EBSLN stimulation, we obtained a mean response value of 19.1 μV from the contralateral CPM and accepted the positive EMG response value as ≥100 μV (24).

Routinely, patients underwent pre- and postoperative (within 2 days) direct laryngoscopy, conducted by an independent laryngologist. Patients who had VCP postoperatively underwent vocal cord examination periodically in the first, second, fourth, and sixth months. RLN palsy was defined as permanent when there was no evidence of recovery within 6 months postoperatively. Since the study was retrospective, postoperative voice quality was not evaluated with either subjective or objective tests.

### Statistical analyses

The incidence of nerve events was calculated based on the number of nerves at risk. All amplitude data are presented as mean and standard deviation (min–max). Positive EMG response values are expressed as frequency and percentage. Differences between continuous and categorical variables were assessed using the Mann–Whitney U test and Fisher’s exact test or chi-square test, respectively. The Wilcoxon signed-rank test was applied for the analysis of the parameters of the two dependent groups.

A value of p < 0.05 was considered to be statistically significant.

## Results

**Figure 4:**
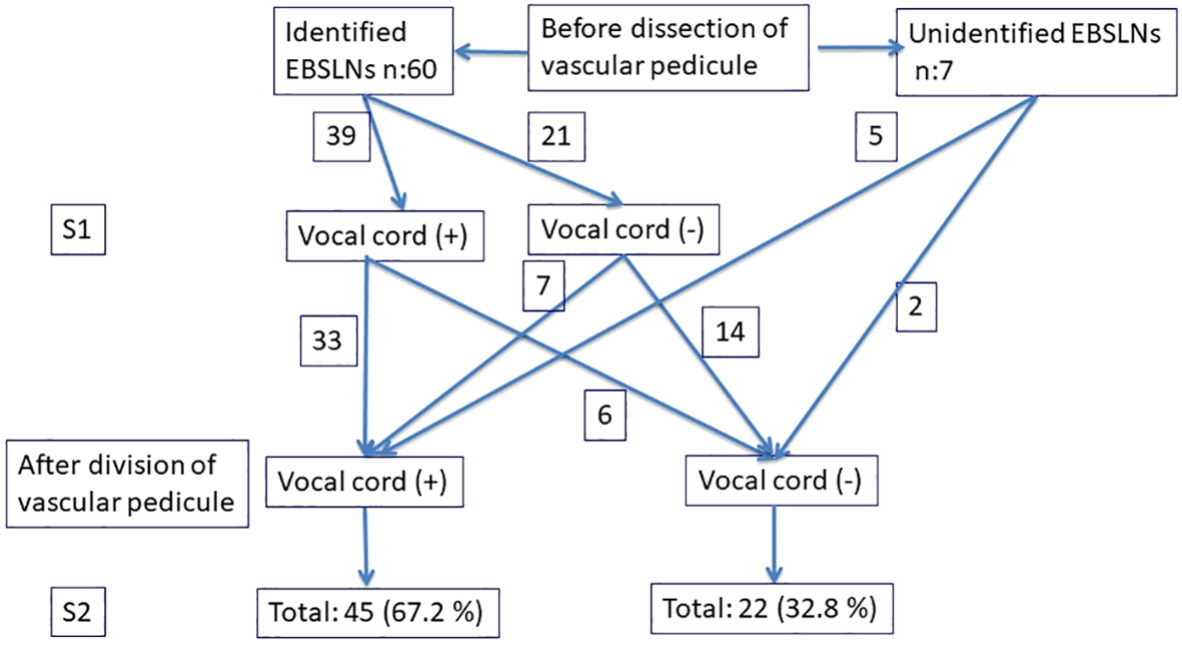
Evaluation of the EBSLNs before and after dividing the vascular pedicle using IONM. EBSLNs, external branch of the superior laryngeal nerves.

The data of 41 patients (32 female and three male) (mean age, 46.7 ± 9.1; range, 22–71) meeting the inclusion criteria were evaluated in the present study. A total of 67 EBSLNs out of 26 bilateral and 15 unilateral interventions were evaluated (**Table 1**). Three patients (4.5%; due to the number of nerves at risk) had unilateral transient VCP. Sixty out of 67 EBSLNs (89.5%) were identified before superior pole dissection using IONM, and the remaining seven (10.5%) were able to be detected using a stimulator probe after the completion of superior pole dissection. CTM twitch combined with positive glottic EMG waveform was achieved in 39 (65%) of 60 EBSLNs, which were detected before superior pole dissection (S1). In six (15.4%) out of 39 EBSLNs, CTM twitch was detected, but no positive glottic EMG response was achieved with EBSLN stimulation after superior pole dissection (S2). After completing superior pole dissection, the positive glottic EMG waveforms were achieved from seven (33.3%) out of 21 EBSLNs, which have been detected before superior pole dissection with CTM twitch but not positive glottic EMG response. Out of the remaining seven EBSLNs identified using a probe after superior pole dissection, five had positive glottic EMG response in addition to the CTM twitch. In two out of three patients with VCP, positive glottic responses were detected with S2 stimulation on the same side as the VCP after achieving S1 and detecting RLN paralysis. Only CTM twitch was observed in one patient with both S1 and S2 stimulation, but no positive response from the vocal cords. With EBSLN stimulation after superior pole dissection, positive glottic EMG waveforms via an endotracheal tube (ETT) were obtained in 45 (67.2%) out of 67 (**Figure 4**), and the mean glottic amplitude value was 261 ± 191 μV (min–max, 116–1,086 μV).

**Table 1:** Demographic profile, surgical indications, and interventions.

	n = 41
**Age (mean** ± **SD) year, (min–max)**	46.7 ± 9.1 (22–71)
**Gender (female/male) n (%)**	32/9 (78/22)
Preoperative diagnosis	
** MNG, n (%)**	26 (63.4)
** Toxic MNG, n (%)**	3 (7.3)
** Graves, n (%)**	2 (4.9)
** Suspicious for malignancy or malignant, n (%)**	10 (24.4)
Surgical intervention, n (%)	26 (63.4)/15 (36.6)
** TT (n)**	23
** TT+CND (n)**	3
** Lobectomy (right/left) (n)**	7/8
**EBSLN at risk (n)**	67

MNG, multinodular goiter; TT, total thyroidectomy; CND, central neck dissection; EBSLN, external branch of the superior laryngeal nerve.

Positive EMG responses via the CTM EMG were achieved from all of the 67 EBSLNs with stimulation using a monopolar probe at the most cranial portion above the area of divided superior pole vessels. The mean value of CTM amplitudes via CTM EMG obtained with EBSLN stimulation was (mean ± SD) 5,268 ± 3,916 μV (min–max, 1,215–19,726 μV). With EBSLN stimulation, the mean CTM EMG amplitude was detected significantly higher than the mean vocal cord amplitude (p < 0.0001). The CTM EMG provided more objective quantifiable data regarding the EBSLN function (100% *vs.* 67.2%, p < 0.001). While applying CTM EMG, no trauma of the muscle such as bleeding or hematoma occurred due to the needle electrode inserted into the muscle.

## Discussion

Recently, postoperative voice quality related to thyroid surgery has been an important issue for both surgeon and patient; hence, awareness of the consequences of EBSLN injury is increasing (18). Although the use of IONM in thyroidectomy is gradually increasing, the routine use of IONM for the EBSLN is not as common as it is for the RLN (25). The proofs regarding the contribution of IONM to the identification and preservation of the EBSLN are gradually increasing (18). Intraoperative EMG of the CTM is an effective method to determine the EBSLN injury and the etiology of the injury (14). Additionally, there are a limited number of studies regarding the intraoperative CTM EMG during thyroidectomy in the literature (14, 26–29).

In this study, we aimed to evaluate whether intraoperative CTM EMG with a needle electrode after thyroidectomy has an informative contribution to the IONM data or not.

In the present study, with the EBSLN stimulation, a positive EMG waveform response was achieved from all the EBSLNs via CTM EMG, and no loss of signal was detected.

Post-dissection positive CTM EMG response provided additional quantitative data in one-third of the nerves with a significant difference compared to the positive glottic response obtained via ETT (100% *vs.* 67.2%, respectively, p < 0.001). The mean amplitude obtained with EBSLN stimulation via CTM EMG was 5,268 ± 3,916 μV (min–max, 1,215–19,726 μV), which was significantly higher than the vocal cord amplitude via ETT (5,268 ± 3,916 μV *vs.* 261 ± 191 μV, p < 0.001). The higher amplitudes may provide more adequate information in evaluating the function of the EBSLN and predicting its prognosis. The higher amplitudes with CTM EMG compared to the amplitudes with ETT are related to the CTM being the target organ of the EBSLN. The TAM is supplied by the RLN, not the EBSLN, and the EBSLN alone may contribute to the anterior third of the TAM via the human communicating nerve.

The main principle of surgery is the identification of an anatomical structure with meticulous dissection so as not to injure it. This rule also applies to the EBSLN because of its variable anatomical course and close relation with the superior thyroid pole (30). However, the EBSLN can be detected functionally using IONM, but the nerve may not be visualized due to its course under the fascia or through the fibers of the IPC muscle (9, 13, 14).

Before superior pole dissection, 89.5% of the EBSLNs were able to be identified due to the CTM twitch assessment. The remaining EBSLNs (10.5%) were able to be identified using IONM after superior pole dissection. During the vessel ligation, the dissected structure was stimulated using a probe and divided in the absence of CTM twitch (negative IONM signal). If the EBSLN cannot be identified during superior pole dissection, a negative IONM signal may be reliable for the surgeon to believe that the dissected structure is not the EBSLN.

With the stimulation of 39 (65%) out of 60 EBSLNs identified before superior pole dissection (S1), the positive glottic response was achieved secondary to the vocal cord innervation of the human communicating nerve in addition to the CTM twitch.

Positive glottic response was achieved with S1 stimulation in two out of three neck sides with postoperative VCP. Although these patients had a loss of signal (LOS) in the RLN, a positive IONM signal similar to the amplitude value of S1 stimulation was achieved with S2 stimulation, which was an important functional indicator for the contribution of the human communicating nerve to the vocal cord innervation.

With S2 stimulation after superior pole dissection, positive glottic response via ETT was achieved from 45 EBSLNs (67.2%) out of 67 including 33 (84.6%) of 39 nerves having positive glottic response with S1 stimulation, seven (33.3%) of 21 nerves having negative glottic response with S1 stimulation, and five of seven nerves detected after superior pole dissection. In 33% of the EBSLNs, with S2 stimulation, quantitative EMG responses were not able to be obtained via ETT regarding the EBSLN function.

The glottic response obtained in addition to the CTM twitch may change before and after superior pole dissection. In six out of 39 EBSLNs with positive EMG glottic response via S1, despite the positive CTM twitch via S2 stimulation, there was a negative glottic EMG response. Although this is primarily thought to be resulting from ETT malpositioning due to the tracheal displacement after tracheal traction and thyroidectomy, the possibility of injury to the human communicating nerve or its intralaryngeal anastomoses or partial injury of the EBSLN cannot be ignored.

Despite the presence of CTM twitch, negative S2 glottic response should not be considered an EBSLN injury. Although CTM twitch is the main positive finding in the EBSLN neuromonitoring via ETT, false-positive CTM twitch can be observed in partial EBSLN injury due to non-neural shunt stimulation via collateral stimulation using a probe within a 2-cm area of the CTM.

Sung et al. (31) prospectively evaluated the diagnostic value of the CTM twitch inspection via postoperative CTM EMG to determine EBSLN integrity in 712 EBSLNs at risk. They found high diagnostic values regarding the CTM inspection for sensitivity and positive predictive value (97.7% and 92.5%, respectively). Despite the low false-negative rate (2.3%), the false-positive rate was high (94.5%), and low specificity (5.5%) and negative predictive value (16.7%) were observed. The authors concluded that CTM twitch observation could be used in determining the EBSLN integrity, but it may not replace EMG considering the low specificity and negative predictive value. The results of this extensive and prospective study are important.

We think that intraoperative CTM EMG can provide important additional informative data on this subject.

There are some limitations of our study. First, it is a retrospective study Although the EBSLN is stimulated at the most cranial portion above the area of the divided superior pole vessels, it cannot be 100% guaranteed whether the nerve is injured more proximally or not. The weak point of this study is that postoperative objective and subjective voice tests have not been performed. To eliminate this, a study should be conducted to prospectively evaluate postoperative voice quality to confirm the anatomical and functional integrity of the EBSLN proved by the use of CTM EMG in the present study. Second, CTM EMG was performed intraoperatively after the thyroidectomy and was not compared with either CTM EMG performed before upper pole dissection or postoperative CTM EMG, which is accepted as a gold standard method. However, as far as we know, this study is the first to evaluate the contribution of intraoperative CTM EMG to the quantitative data obtained via IONM with ETT. It is important to obtain quantitative positive EMG recordings from the CTM in all of the EBSLNs, to be able to store them, and to have high EMG amplitude values in terms of the EBSLN function. CTM EMG is a simple, safe, and applicable method for surgeons using IONM.

In conclusion, in addition to the IONM via ETT, intraoperative post-dissection CTM EMG via a needle electrode is a safe, simple, and applicable method. It is a method that may provide significant additional informative data to IONM with ETT by obtaining and recording objective quantitative data related to the EBSLN function.

## Data availability statement

The original contributions presented in the study are included in the article/supplementary material. Further inquiries can be directed to the corresponding author.

## Author contributions

NA: Conceptualization, Investigation, Writing – original draft, Writing – review & editing. MU: Conceptualization, Data curation, Investigation, Methodology, Writing – review & editing. MK: Data curation, Investigation, Methodology, Writing – review & editing. OC: Data curation, Investigation, Methodology, Writing – review & editing. AI: Supervision, Writing – review & editing. MU: Conceptualization, Investigation, Supervision, Writing – review & editing.

## References

[1] Nam IC Park YH. Pharyngolaryngeal symptoms associated with thyroid disease. Curr Opin Otolaryngol Head Neck Surg (2017) 25:469–74. doi: 10.1097/MOO.0000000000000404 28759458

[2] Zealear DL Billante CR. Neurophysiology of vocal fold paralysis. Otolaryngol Clin N Am (2004) 37:1–23. doi: 10.1016/S0030-6665(03)00165-8 15062684

[3] Wu BL Sanders I Mu L Biller HF. The human communicating nerve. An extension of the external superior laryngeal nerve that innervates the vocal cord. Arch Otolaryngol Head Neck Surg (1994) 120:1321–8. doi: 10.1001/archotol.1994.01880360019004 7980895

[4] Maranillo E Leon X Quer M Orus C Sanudo JR. Is the external laryngeal nerve an exclusively motor nerve? The cricothyroid connection branch. Laryngoscope (2003) 113:525–9. doi: 10.1097/00005537-200303000-00024 12616208

[5] Cernea CR AR F Nishio S Dutra A Jr FC H dos Santos LR. Surgical anatomy of the external branch of the superior laryngeal nerve. Head Neck (1992) 14(5):380–3. doi: 10.1002/hed.2880140507 1399571

[6] Cheruiyot I Kipkorir V Henry BM Munguti J Cirocchi R Odula P. Surgical anatomy of the external branch of the superior laryngeal nerve: a systematic review and meta-analysis. Langenbecks Arch Surg (2018) 403(7):811–23. doi: 10.1007/s00423-018-1723-9 30430230

[7] Feng AL Puram SV Singer MC Modi R Kamani D Randolph GW. Increased prevalence of neural monitoring during thyroidectomy: global surgical survey. Laryngoscope (2020) 130:1097–104. doi: 10.1002/lary.28210 31361342

[8] Randolph GW Dralle H International Intraoperative Monitoring Study Group Abdullah H Barczynski M Bellantone R. Electrophysiologic recurrent laryngeal nerve monitoring during thyroid and parathyroid surgery: international standards guideline statement. Laryngoscope (2011) 121:S1–16. doi: 10.1002/lary.21119 21181860

[9] Barczyński M Randolph GW Cernea CR Dralle H Dionigi G Alesina PF. External branch of the superior laryngeal nerve monitoring during thyroid and parathyroid surgery: International Neural Monitoring Study Group standards guideline statement. Laryngoscope (2013) 123:S1–14. doi: 10.1002/lary.24301 23832799

[10] Bellantone R Boscherini M Lombardi CP Bossola M Rubino F De Crea C. Is the identification of the external branch of the superior laryngeal nerve mandatory in thyroid operation? Results of a prospective randomized study. Surgery (2001) 130:1055–9. doi: 10.1067/msy.2001.118375 11742338

[11] Lennquist S Cahlin C Smeds S. The superior laryngeal nerve in thyroid surgery. Surgery (1987) 102:999–1008. 3686359

[12] Cernea CR Ferraz AR Furlani J Monteiro S Nishio S Hojaij FC. Identification of the external branch of the superior laryngeal nerve during thyroidectomy. Am J Surg (1992) 164:634–9. doi: 10.1016/S0002-9610(05)80723-8 1463114

[13] Uludag M Aygun N Kartal K Besler E Isgor A. Is intraoperative neural monitoring necessary for exploration of the superior laryngeal nerve? Surgery (2017) 161:1129–38. doi: 10.1016/j.surg.2016.10.026 27989608

[14] Uludag M Aygun N Kartal K Çitgez B Besler E Yetkin G. Contribution of intraoperative neural monitoring to preservation of the external branch of the superior laryngeal nerve: A randomized prospective clinical trial langenbecks arch surg. Langenbeck's Archives of Surgery (2017) 402:965–76. doi: 10.1007/s00423-016-1544-7 28035477

[15] Barczyński M Konturek A Stopa M Honowska A Nowak W. Randomized controlled trial of visualization versus neuromonitoring of the external branch of the superior laryngeal nerve during thyroidectomy. World J Surg (2012) 36:1340–7. doi: 10.1007/s00268-012-1547-7 PMC3348444 22402975

[16] Potenza AS Phelan EA Cernea CR Slough CM Kamani DV Darr A. Normative intra-operative electrophysiologic waveform analysis of superior laryngeal nerve external branch and recurrent laryngeal nerve in patients undergoing thyroid surgery. World J Surg (2013) 37:2336–42. doi: 10.1007/s00268-013-2148-9 23838931

[17] Grover G Sadler GP Mihai R. Morbidity after thyroid surgery: patient perspective. Laryngoscope (2013) 123:2319–23. doi: 10.1002/lary.23850 23824630

[18] Potenza AS Araujo Filho VJF Cernea CR. Injury of the external branch of the superior laryngeal nerve in thyroid surgery. Gland Surg (2017) 6:552–62. doi: 10.21037/gs.2017.06.15 PMC5676167 29142848

[19] Dralle H Lorenz K Machens A. Verdicts on malpractice claims after thyroid surgery: emerging trends and future directions. Head Neck (2012) 34:1591–6. doi: 10.1002/hed.21970 22431167

[20] Uludag M Aygun N Isgor A. Motor function of the recurrent laryngeal nerve: sometimes motor fibers are also located in the posterior branch. Surgery (2016) 160:153–60. doi: 10.1016/j.surg.2016.02.003 26972775

[21] Aygün N Uludağ M İşgör A. Contribution of intraoperative neuromonitoring to the identification of the external branch of superior laryngeal nerve. Turk J Surg (2017) 33:169–74. doi: 10.5152/turkjsurg.2017.3645 PMC5602307 28944328

[22] Dionigi G Kim HY Randolph GW Wu CW Sun H Liu X. Prospective validation study of Cernea classification for predicting EMG alterations of the external branch of the superior laryngeal nerve. Surg Today (2016) 46:785–91. doi: 10.1007/s00595-015-1245-9 26362419

[23] Martin-Oviedo C Maranillo E Lowy-Benoliel A Pascual-Font A Martinez-Guirado T Rodriguez-Niedenführ M. Functional role of human laryngeal nerve connections. Laryngoscope (2011) 21:2338–43. doi: 10.1002/lary.22340 21919010

[24] Uludag M Aygun N Isgor A. Innervation of the human cricopharyngeal muscle by the recurrent laryngeal nerve and external branch of the superior laryngeal nerve. Langenbeck’s Arch Surg (2017) 402:683–90. doi: 10.1007/s00423-016-1376-5 26843022

[25] Barczyński M Randolph GW Cernea C. International Neural Monitoring Study Group in Thyroid and Parathyroid Surgery. International survey on the identification and neural monitoring of the EBSLN during thyroidectomy. Laryngoscope (2016) 126:285–91. doi: 10.1002/lary.25548 26452247

[26] Liddy W Barber SR Cinquepalmi M Lin BM Patricio S Kyriazidis N. The electrophysiology of thyroid surgery: electrophysiologic and muscular responses with stimulation of the vagus nerve, recurrent laryngeal nerve, and external branch of the superior laryngeal nerve. Laryngoscope (2017) 127:764–71. doi: 10.1002/lary.26147 27374859

[27] Selvan B Babu S Paul MJ Abraham D Samuel P Nair A. Mapping the compound muscle action potentials of cricothyroid muscle using electromyography in thyroid operations: a novel method to clinically type the external branch of the superior laryngeal nerve. Ann Surg (2009) 250:293–300. doi: 10.1097/SLA.0b013e3181b17342 19638904

[28] Gavid M Dubois MD Larivé E Prades JM. Superior laryngeal nerve in thyroid surgery: anatomical identification and monitoring. Eur Arch Otorhinolaryngol (2017) 274:3519–26. doi: 10.1007/s00405-017-4666-9 28687919

[29] Iwata AJ Liddy W Barczyński M Wu CW Huang TY Van Slycke S. Superior laryngeal nerve signal attenuation influences voice outcomes in thyroid surgery laryngoscope. Laryngoscope (2021) 131:1436–42. doi: 10.1002/lary.29413 33521945

[30] Patnaik U Nilakantan A Shrivastava T. Anatomical variations of the external branch of the superior laryngeal nerve in relation to the inferior constrictor muscle: cadaveric dissection study. J Laryngol Otol (2012) 126:907–12. doi: 10.1017/S0022215112001454 22784932

[31] Sung ES Chang JH Kim J Cha W. Is cricothyroid muscle twitch predictive of the integrity of the EBSLN in Thyroid Surgery? Laryngoscope (2018) 128:2654–61. doi: 10.1002/lary.27158 29573416

